# CASP8 and CASP3 mRNA Expression in Autoimmune Lymphoproliferative Syndrome (ALPS) and Chronic Immune Thrombocytopenia (ITP)

**DOI:** 10.3390/genes17020206

**Published:** 2026-02-09

**Authors:** Anna Pau, Federico Rondot, Stefano Gambarino, Anna Clemente, Cristina Calvi, Paola Montanari, Ilaria Galliano, Massimiliano Bergallo

**Affiliations:** 1 Immunopathology Laboratory, Department of Public Health and Pediatric Sciences, Medical School, University of Turin, Piazza Polonia, 94, 10126 Turin, Italy; pauanna1@gmail.com (A.P.); stefano.gambarino@unito.it (S.G.); anna.clemente@unito.it (A.C.); cristina.calvi@unito.it (C.C.); paola.montanari@unito.it (P.M.); 2 Department of Molecular Biotechnology and Health Sciences, University of Turin, 10126 Turin, Italy; federico.rondot@unito.it

**Keywords:** apoptosis, CASP8, CASP3, autoimmune lymphoproliferative syndrome (ALPS), immune thrombocytopenia (ITP), lymphocyte homeostasis

## Abstract

Background: Fas/FasL-mediated apoptosis is central to immune homeostasis and is implicated in autoimmune lymphoproliferative syndrome (ALPS) and immune thrombocytopenia (ITP). We aimed to compare whole-blood transcriptional levels of CASP8 and CASP3 across ALPS, chronic ITP, and healthy controls. Methods: CASP8 and CASP3 mRNA expression was quantified by real-time PCR in whole blood from clinically diagnosed ALPS patients, chronic ITP patients, and healthy controls. Results: CASP8 mRNA expression was significantly increased in ALPS and ITP versus controls (*p* = 0.0009 and *p* < 0.0001, respectively) and was lower in ALPS than in ITP (*p* = 0.0265). CASP3 mRNA was also increased in both patient groups versus controls (ALPS: *p* = 0.0045; ITP: *p* < 0.0001), with no significant difference between ALPS and ITP (*p* = 0.1692). Conclusions: ALPS and chronic ITP show distinct CASP8 transcriptional patterns and a shared upregulation of CASP3 at the whole-blood mRNA level. These findings are descriptive and do not directly assess caspase activation or apoptotic pathway activity; further protein- and cell subset-based studies are needed to clarify functional implications.

## 1. Introduction

Apoptosis, or programmed cell death, is essential for immune homeostasis by regulating lymphocyte expansion and removal. Physiological apoptosis prevents autoimmunity and supports flexible immune response through the elimination of self-reactive, damaged, or redundant lymphocytes [1]. A key regulator of extrinsic apoptosis in activated immune cells is the Fas/Fas ligand (FasL) pathway [2]. Fas engagement triggers downstream signaling that can involve initiator caspases such as caspase-8 and executioner caspases such as caspase-3, ultimately leading to apoptotic cell death [3,4].

Defects in the Fas/FasL pathway lead to autoimmune and lymphoproliferative defects. One example of those defects is the autoimmune lymphoproliferative syndrome (ALPS), which is mainly caused by heterozygous germline or somatic mutations in the *FAS* gene (ALPS-FAS/ALPS-sFAS) [5,6,7] or in other components of the extrinsic apoptosis pathway, such as *FASLG* (ALPS-FASL) and *CASP10* (ALPS-CASP10) [8,9,10]. These mutations are associated with impaired Fas-mediated apoptotic signaling and altered downstream activation of caspase pathways. The main clinical features of ALPS are chronic lymphoproliferation (lymphadenopathy and splenomegaly) and a predisposition to autoimmune cytopenias and lymphomas [11,12]. The hallmark immunological feature of ALPS patients is the accumulation of CD3^+^ CD4^−^ CD8^−^ “double-negative” T cells (DNTs), a direct sign of failed apoptosis [13].

Despite strong evidence of genetic bases of ALPS, at least half of the patients fulfill the diagnostic criteria for ALPS but cannot be genetically diagnosed and are classified as ALPS undetermined (ALPS-U), according to the ALPS classification proposed in 2010 [12,14]. In addition, ALPS demonstrates a significant phenotypic overlap with Evans syndrome, defined by the presence of at least two autoimmune cytopenias without lymphoproliferation, highlighting that a subgroup of patients may not present the classic lymphoproliferative signs [15]. In this context, descriptive transcriptional profiling of apoptosis-related genes may provide complementary information to support clinical interpretation in diagnostically challenging cases.

Immune thrombocytopenia (ITP) is an autoimmune disorder characterized by antibody-mediated platelet destruction and impaired platelet production [16]. Although it is not considered a monogenic condition, several studies suggest that Fas/FasL-related immune regulation and apoptosis may be altered in ITP, including changes in Fas/FasL expression in peripheral blood [17,18,19,20].

Therefore, we performed a comparative analysis of CASP8 and CASP3 mRNA expression in whole blood from patients with clinically diagnosed ALPS, chronic ITP, and healthy controls. This short communication aims to describe transcriptional patterns of these apoptosis-related genes across the three groups. Protein expression, caspase activation, and cell subset-specific effects were not assessed in this study.

## 2. Materials and Methods

### 2.1. Patient Selection

Whole-blood samples were collected in EDTA-treated tubes from patients with a clinical diagnosis of ALPS (defined by non-malignant, non-reactive chronic lymphoproliferation clinically determined by the clinical detection of lymphadenopathy and/or splenomegaly persisting for ≥6 months and a percentage of DNT cells > 2.5% within the CD3^+^ T-cell population), from children with chronic ITP (thrombocytopenia lasting >12 months with no clinical lymphoproliferation or increase in DNT cells), and from healthy controls (HCs). A total of 23 individuals with ALPS were included, 8 of whom had previously received immunomodulatory therapy (mycophenolate mofetil or sirolimus) for the management of lymphoproliferation and cytopenias. The ITP group comprised 27 children, none of whom exhibited clinical or laboratory features suggestive of ALPS. Lastly, 22 healthy controls (HCs) were selected from asymptomatic individuals undergoing routine laboratory testing at the Regina Margherita Children’s Hospital (Turin, Italy), all of whom had results within normal reference ranges. Subjects meeting exclusion criteria (including infections, cancer, autoimmune or inflammatory disorders, neurological diseases, or abnormal laboratory findings) were excluded prior to analysis, and no additional individuals were enrolled beyond those reported. All analyses were performed on leftover clinical samples after informed parental consent, and data were gathered anonymously. The study was conducted in accordance with institutional and national ethical guidelines and with the principles of the 1964 Helsinki Declaration and its later amendments; informed consent was obtained from all participants or their legal guardians.

### 2.2. Reverse Transcription

Total RNA was extracted from whole blood using the automated extractor Maxwell (Promega, Madison, WI, USA) using the RNA Blood Kit protocol without modification. This kit provides treatment with DNase during the RNA extraction process. Afterwards, the RNA concentration was quantified by the NanoDrop 2000™ (Thermo Fisher Scientific, Waltham, MA, USA). Then, 500 ng of total RNA was reverse-transcribed with 2 μL of buffer 10×, 4.8 μL of MgCl2 (25 mM), 2 μL of Improm-II (Promega), 1 μL of RNase inhibitor 20 U/L, 0.4 μL of random hexamers (250 μM; Promega), 2 μL of mixed dNTPs (100 mM) (Promega), and dd-water in a final volume of 20 μL. The reaction mix was carried out in a GeneAmp PCR system 9700 Thermal Cycle (Applied Biosystems, Foster City, CA, USA) under the following conditions: 5 min at 25 °C, 60 min at 42 °C, and 15 min at 70 °C for the inactivation of the enzyme; the cDNAs were stored at −80° until use. All these procedures were performed according to the manufacturers’ protocols. Regarding control for genomic DNA contamination, we directly amplified RNA extract without reverse transcription.

### 2.3. Relative Quantification of CASP3 and CASP8

Relative quantification of CASP3 and CASP8 transcripts was achieved by means of PCR real-time Taqman amplification and normalization to glyceraldehyde-3-phosphate dehydrogenase (GAPDH), which was chosen as a reference gene, using the ABI PRISM 7500 real-time system (Life Technologies, Austin, TX, USA). Then, 50 ng of cDNA were amplified in a 20 μL total volume reaction containing Go-Taq mastermix probe (Promega), 500 nmol of specific primers, and 200 nmol of specific probes. The primers and probes used were reported in Table 1:

The established assays use probes and primers designed by Primer Express TM software version 3.0 (Applied Biosystems, Foster City, CA, USA). Basic Local Alignment Search Tool (BLAST, Version 2.12.0) analysis confirmed no cross-reaction. The amplifications were run on a 96-well plate at 95 °C for 2 min, followed by 45 cycles at 95 °C for 15 s and at 60 °C for 1 min. Relative quantification of target gene expression was performed with the ΔΔCt and RQ method.

### 2.4. Statistical Analysis

Data distribution was assessed using the Shapiro–Wilk test. As CASP8 and CASP3 mRNA expression values did not follow a normal distribution, non-parametric statistical methods were applied. Comparisons among ALPS, chronic ITP, and healthy control groups were performed using the Kruskal–Wallis test, followed by Dunn’s multiple comparisons test. A *p*-value < 0.05 was considered statistically significant. All statistical analyses were performed using GraphPad Prism software (Version 7, GraphPad Software, La Jolla, CA, USA).

## 3. Results

### 3.1. Study Population

Demographic characteristics of subjects enrolled in this study are detailed in Table 2.

### 3.2. CASP8 mRNA Expression

**Figure 1:**
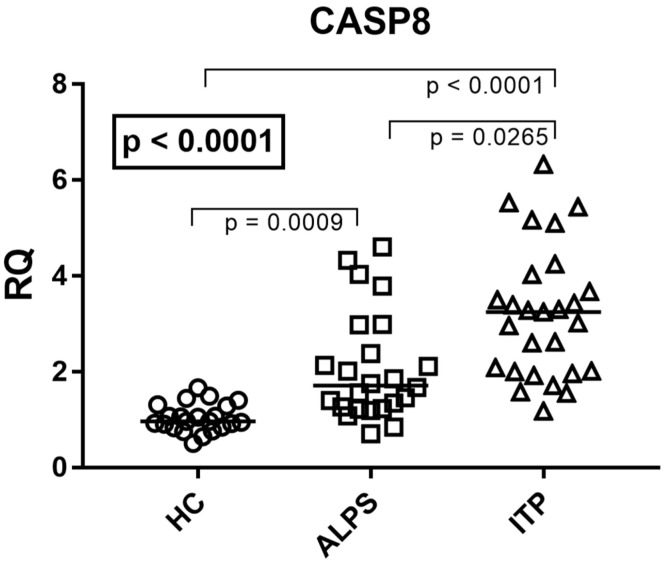
*CASP8* mRNA expression is differentially upregulated in ITP and ALPS. Representative blot image of *CASP8* mRNA expression in whole blood from healthy controls (HCs), immune thrombocytopenia (ITP) patients, and autoimmune lymphoproliferative syndrome (ALPS) patients. Analysis of *CASP8* mRNA expression was normalized to GAPDH expression. Data are presented as RQ (relative quantification) calculated with the ΔΔCt method. Horizontal lines represent the median values. Statistical analysis: Kruskal–Wallis test (boxed *p* values), followed by Dunn’s multiple comparisons test.

To evaluate the status of the initiator caspase in the extrinsic apoptotic pathway, we quantified the mRNA expression levels of *CASP8* in whole blood in all three different groups. Median and IQR (25–75%) were 0.97, 0.87–1.24 for HC; 1.76, 1.31–2.68 for ALPS; and 3.25, 1.99–3.85 for ITP. A highly significant upregulation of *CASP8* was observed in both patient cohorts compared to healthy individuals (*p* = 0.0009 for ALPS and *p* < 0.0001 for ITP vs. HC) (Figure 1). Notably, a direct comparison between the two disease groups revealed a statistically significant difference, with *CASP8* levels significantly lower in ALPS patients compared to ITP patients (*p* = 0.0265) (Figure 1).

### 3.3. CASP3 mRNA Expression

**Figure 2:**
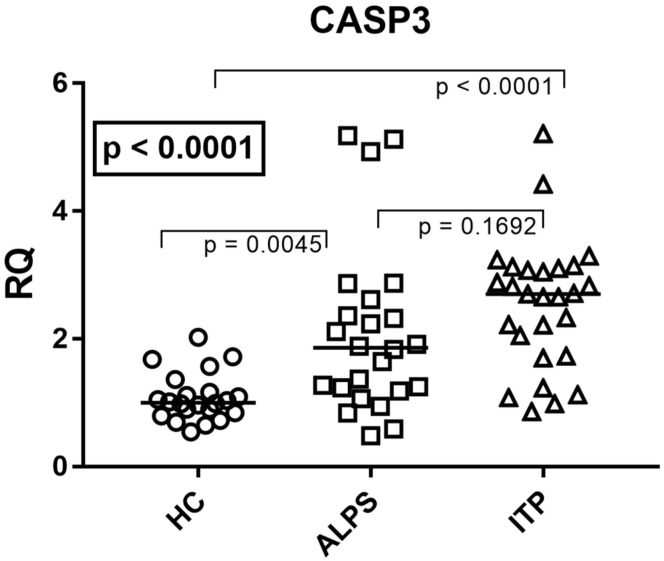
*CASP3* mRNA expression is upregulated in ITP and ALPS without a significant inter-group difference. Representative blot image of *CASP3* expression in whole blood from healthy controls (HCs), immune thrombocytopenia (ITP) patients, and autoimmune lymphoproliferative syndrome (ALPS) patients. Analysis of *CASP3* expression was normalized to GAPDH expression. Data are presented as RQ (relative quantification) calculated with the ΔΔCt method. Horizontal lines represent the median values. Statistical analysis: Kruskal–Wallis test (boxed *p* values), followed by Dunn’s multiple comparisons test.

We next analyzed the expression of the key executioner *CASP3*, which acts downstream of both the extrinsic and intrinsic apoptotic pathways. Median and IQR (25–75%) were 1.00, 0.86–1.15 for HC; 1.89, 1.24–2.49 for ALPS; and 3.25, 2.68, 1.81–3.09 for ITP. Consistent with the upstream findings, *CASP3* levels were also significantly elevated in whole blood from both ITP and ALPS patients compared to HCs (*p* = 0.0045 for ALPS and *p* < 0.0001 for ITP vs. HC) (Figure 2). *CASP3* levels showed no differences between ITP and ALPS patients (*p* = 0.1692) (Figure 2).

Individual CASP8 and CASP3 mRNA expression values for all subjects are provided in Supplementary Table S1.

## 4. Discussion

The present study provides a comparative description of whole-blood CASP8 and CASP3 mRNA expression in ALPS and chronic ITP. Overall, both patient groups showed significantly increased CASP8 and CASP3 transcript levels compared with healthy controls. Notably, CASP8 mRNA levels were lower in ALPS than in chronic ITP, whereas CASP3 levels did not differ significantly between the two diseases. These observations indicate transcriptional differences across the cohorts and do not directly address caspase activation or functional apoptotic responses [1,2,3,4].

The increased *CASP8* mRNA levels observed in both ALPS and chronic ITP compared with healthy controls are consistent with prior reports indicating that components of Fas/FasL-related signaling can be transcriptionally modulated in immune dysregulation [1,2,3,17,18,19,20]. Importantly, *CASP8* transcription levels were significantly lower in ALPS than in chronic ITP. At the whole-blood mRNA level, this difference highlights a distinct transcriptional pattern between the two conditions; however, it should not be interpreted as a direct measure of caspase-8 activation or pathway functionality. Indeed, Fas-mediated apoptosis is regulated at multiple post-transcriptional and post-translational levels, including DISC assembly and proteolytic processing of initiator caspases [3,4]. Future studies integrating protein-level readouts and analyses in defined immune cell subsets will be required to clarify the biological and clinical significance of CASP8 transcriptional differences.

*CASP3* mRNA expression was similarly increased in both ALPS and chronic ITP compared with controls, without a significant difference between the two diseases. Caspase-3 represents a central executioner of apoptosis downstream of both extrinsic and intrinsic pathways [4]. However, caspase activity depends primarily on proteolytic cleavage and enzymatic activation rather than transcript abundance alone. Therefore, the observed CASP3 transcriptional upregulation cannot be directly translated into functional apoptotic activity, and protein-level analyses, such as detection of cleaved caspase-3, are required to assess pathway activation [4,21,22].

Although ITP is not considered a monogenic disorder affecting core apoptotic machinery, previous studies have reported altered expression of Fas/FasL-related markers and caspase-associated transcripts in the peripheral blood of ITP patients [17,18,19,20]. Increased caspase-3 expression has also been described in pediatric ITP cohorts [23]. In line with this literature, our data show increased CASP8 and CASP3 mRNA levels in chronic ITP compared with healthy controls, supporting the involvement of apoptosis-related transcriptional changes in this condition.

Although ITP is not considered a monogenic disorder affecting core apoptotic machinery, previous studies have reported altered expression of Fas/FasL-related markers and caspase-associated transcripts in the peripheral blood of ITP patients [19,20]. In addition, increased caspase-3 expression has been described in newly diagnosed and pediatric ITP cohorts [23]. In line with these observations, our findings show increased CASP8 and CASP3 mRNA levels in chronic ITP patients compared with healthy controls, supporting the presence of apoptosis-related transcriptional changes in this condition.

Taken together, these data indicate overlapping but distinct transcriptional features of CASP8 and CASP3 in ALPS and chronic ITP at the whole-blood mRNA level. While both conditions show increased transcript levels compared with controls, the lower CASP8 expression observed in ALPS suggests a disease-specific transcriptional profile. These observations are hypothesis-generating and provide a rationale for future studies integrating genetic background, immune cell subset analyses, and protein-level measurements to clarify functional and clinical implications [12,14].

Exploratory stratification of ALPS patients according to immunomodulatory treatment status did not reveal significant differences in CASP8 or CASP3 mRNA expression.

This study has limitations inherent to its descriptive design. Analyses were restricted to whole-blood mRNA expression and did not include protein abundance, caspase activation, or functional apoptosis assays. In addition, cell subset-specific effects, including those involving double-negative T cells, were not assessed. The ALPS cohort was defined on clinical criteria, and systematic genetic characterization was not available for all patients. Finally, heterogeneity related to treatment exposure and disease duration may have contributed to inter-individual variability. In addition, the sample size was limited, and the cohort was derived from a single geographical region, which may restrict the generalizability of the present findings. These limitations should be considered when interpreting the present findings.

## 5. Conclusions

In this short communication, we report increased CASP8 and CASP3 mRNA expression in whole blood from patients with autoimmune lymphoproliferative syndrome (ALPS) and chronic immune thrombocytopenia (ITP) compared with healthy controls, with lower CASP8 transcript levels in ALPS than in ITP. These findings describe transcriptional differences at the whole-blood level and do not directly provide information on caspase activation or apoptotic pathway functionality. Further studies integrating genetic, cellular, and protein-level analyses will be required to clarify their biological and clinical relevance.

## Figures and Tables

**Table 1:** Primers and Probes used for amplification.

Target		Primers	Probes
GAPDH	PF	5′-CGAGATCCCTCCAAAATCAA-3′	6FAM-TGGAGAAGGCTGGGGCTCAT-TAMRA
	PR	5′-TTCACACCCATGACGAACAT-3′	
CASP3	PF	5′-TGCGCTGCTCTGCCTTCT-3′	6FAM-TGGAGAAGGCTGGGGCTCAT-TAMRA
	PR	5′-CCATGGGTAGCAGCTCCTTC-3′	
CASP8	PF	5′-AAGTGCCCAAACTTCACAGC-3′	6FAM-ACTTGGATGCAGGGGCTTTGACCAC-TAMRA
	PR	5′-GGGGCTTGATCTCAAAATGA-3′	

**Table 2:** Demographic characteristics of the enrolled subjects.

Characteristic	ALPS (n = 23)	ITP (n = 27)	HC (n = 22)
Sex: male n (%)	15 (65.2)	13 (48.1)	14 (63.6)
Age: median (IQR 25–75%)	15.9 (13.2–28.1)	12.4 (8.9–15.3)	11.7 (8.4–17.0)

ALPS = autoimmune lymphoproliferative syndrome; ITP = immune thrombocytopenia; HC = healthy control; n = number; % = percentage; IQR = interquartile range.

## Data Availability

The data presented in this study are available on request from the corresponding authors (the data are not publicly available due to privacy restrictions).

## Supplementary Materials

The following supporting information can be downloaded at https://www.mdpi.com/article/10.3390/genes17020206/s1. Table S1: Individual CASP8 and CASP3 mRNA expression values (relative quantification, RQ) for all subjects included in the study.
