# Supplementary material for: CASP8 and CASP3 mRNA Expression in Autoimmune Lymphoproliferative Syndrome (ALPS) and Chronic Immune Thrombocytopenia (ITP)
Source: Genes (Basel). 2026 Feb 9;17(2):206. doi: 10.3390/genes17020206 (PMC12940764; doi:10.3390/genes17020206)
Supplement: Supplementary file 1 [file genes-17-00206-s001.zip › genes-4048111-supplementary.pdf]

**Supplementary Table S1.** Individual CASP8 and CASP3 mRNA expression values (relative quantification, RQ) for all subjects included in the study. Data are shown for autoimmune lymphoproliferative syndrome (ALPS), chronic immune thrombocytopenia (ITP) and healthy controls (HC).

| Subject ID | Group | CASP8 mRNA RQ | CASP3 mRNA RQ |
|---|---|---|---|
| S01 | ALPS | 2.115146 | 0.945252 |
| S02 | ALPS | 1.465018 | 2.361009 |
| S03 | ALPS | 2.993112 | 2.11773 |
| S04 | ALPS | 1.855353 | 1.838274 |
| S05 | ALPS | 1.196905 | 0.485189 |
| S06 | ALPS | 1.221074378 | 0.842864 |
| S07 | ALPS | 2.380142 | 1.888228 |
| S08 | ALPS | 4.330286 | 4.931914 |
| S09 | ALPS | 3.79028 | 5.12637 |
| S10 | ALPS | 2.980594 | 5.181425 |
| S11 | ALPS | 0.708095 | 1.251611 |
| S12 | ALPS | 1.679101 | 1.370446 |
| S13 | ALPS | 0.853285 | 1.069316 |
| S14 | ALPS | 1.236261 | 0.591096 |
| S15 | ALPS | 1.755343 | 1.276882 |
| S16 | ALPS | 2.013952 | 2.237499 |
| S17 | ALPS | 2.136865 | 2.615671 |
| S18 | ALPS | 1.405604 | 1.917911 |
| S19 | ALPS | 1.56597 | 1.64679 |
| S20 | ALPS | 1.271858 | 1.23591 |
| S21 | ALPS | 1.08598 | 2.323881 |
| S22 | ALPS | 1.349234 | 1.188623 |
| S23 | ALPS | 4.037874 | 2.86921 |
| S24 | ALPS | 4.611563 | 2.871741 |
| S25 | ITP | 3.43220792 | 2.22116771 |
| S26 | ITP | 5.16815118 | 2.712345 |
| S27 | ITP | 4.02698771 | 3.1264807 |
| S28 | ITP | 3.24844278 | 2.70085128 |
| S29 | ITP | 1.9618672 | 0.86410628 |
| S30 | ITP | 3.3024305 | 3.10088845 |
| S31 | ITP | 2.60356376 | 0.98810181 |
| S32 | ITP | 1.92187385 | 2.33310108 |
| S33 | ITP | 3.50342889 | 5.2116471 |
| S34 | ITP | 1.18107677 | 1.12523316 |
| S35 | ITP | 3.67448685 | 2.65598884 |
| S36 | ITP | 1.71902641 | 1.70355188 |

| | | | |
|---|---|---|---|
| S37 | ITP | 6.32763006 | 3.14940155 |
| S38 | ITP | 1.58070513 | 2.05284556 |
| S39 | ITP | 1.5566154 | 1.23154323 |
| S40 | ITP | 2.62086593 | 3.07034208 |
| S41 | ITP | 4.24882266 | 3.04579318 |
| S42 | ITP | 5.44047836 | 3.29584098 |
| S43 | ITP | 2.00379145 | 1.08392654 |
| S44 | ITP | 5.10398408 | 2.6580649 |
| S45 | ITP | 3.39451361 | 2.21811287 |
| S46 | ITP | 3.02186708 | 2.83110349 |
| S47 | ITP | 5.52994246 | 4.42258128 |
| S48 | ITP | 2.01746946 | 2.8206203 |
| S49 | ITP | 2.09239452 | 1.73209655 |
| S50 | ITP | 2.96642618 | 2.88238765 |
| S51 | ITP | 3.28598534 | 3.23813368 |
| S52 | HC | 1.407299849 | 0.904643829 |
| S53 | HC | 1.671280326 | 2.026580547 |
| S54 | HC | 0.759996792 | 0.5477794 |
| S55 | HC | 0.972671245 | 1.367604764 |
| S56 | HC | 1.057300523 | 0.848333639 |
| S57 | HC | 1.319753826 | 1.017307772 |
| S58 | HC | 0.948908901 | 1.03522658 |
| S59 | HC | 0.834069542 | 1.052446182 |
| S60 | HC | 0.908633829 | 0.991242009 |
| S61 | HC | 1.066410054 | 0.900540102 |
| S62 | HC | 0.920389273 | 0.795835686 |
| S63 | HC | 0.771000573 | 1.098575637 |
| S64 | HC | 0.927910595 | 0.967326471 |
| S65 | HC | 0.972178854 | 1.72062537 |
| S66 | HC | 1.288576566 | 1.166820466 |
| S67 | HC | 1.077676224 | 0.651957341 |
| S68 | HC | 0.508675923 | 0.72687849 |
| S69 | HC | 1.496686585 | 1.117583054 |
| S70 | HC | 1.44788718 | 1.571119874 |
| S71 | HC | 1.074605959 | 0.988946065 |
| S72 | HC | 0.851109284 | 1.683190595 |
| S73 | HC | 0.648301618 | 0.698914293 |
